# Baseline expression levels of *WNT8B*, *KRT2*, and *TTLL13P* are associated with abrocitinib response in atopic dermatitis

**DOI:** 10.1016/j.jdin.2025.10.007

**Published:** 2025-10-22

**Authors:** Yuwen Gao, Yang Luo, Yingxia Gao, Xingyu Chen, Yu Zhang, Yuan Zhou, Beilei Xu, Xu Yao, Xiaochun Liu

**Affiliations:** Jiangsu Provincial Key Laboratory of Dermatololgy, Department of Allergy and Rheumatology, Hospital for Skin Diseases, Institute of Dermatology, Chinese Academy of Medical Sciences & Peking Union Medical College, Nanjing, Jiangsu, China

**Keywords:** abrocitinib, atopic dermatitis, biomarkers, RNA sequencing, treatment response

*To the Editor:* Atopic dermatitis (AD) is a heterogeneous inflammatory skin disease with marked variability in treatment response.^1^ Although the Janus Kinase 1 (JAK1) inhibitor abrocitinib was effective in clinical trials,^2^ real-world biomarkers predicting treatment response remain underexplored.^3^^,^^4^ This study aimed to identify serum and transcriptomic biomarkers associated with treatment response to abrocitinib.

We conducted a real-world study involving 96 patients with moderate-to-severe AD (aged ≥12 years), treated with abrocitinib for 12 weeks (Supplementary Table I, available via Mendeley at https://data.mendeley.com/datasets/g6hvm2znjn/1). Clinical assessments and specimens collection were performed at baseline and week 12 (Supplementary Fig 1, available via Mendeley at https://data.mendeley.com/datasets/g6hvm2znjn/1). Peripheral blood mononuclear cells (PBMCs) and serum were analyzed using RNA sequencing and multiplex immunoassays, respectively. Healthy controls (HCs) were included to establish comparative immune and transcriptomic profiles (Supplementary Figs 2 and 3, available via Mendeley at https://data.mendeley.com/datasets/g6hvm2znjn/1). Detailed methods are described in the Supplementary Materials, available via Mendeley at https://data.mendeley.com/datasets/g6hvm2znjn/1.

Based on the Aiming High in Eczema/Atopic Dermatitis recommendations, which propose establishing minimal disease activity (MDA) as a novel approach to AD management^5^ and for identifying biomarkers linked to optimal response under this framework, patients were divided into 2 groups, EASI 90_yes and EASI 90_no, according to whether they achieved EASI-90 after 12 weeks of treatment. Serum analyses revealed EASI 90_yes patients had lower baseline levels of Th2 cytokines (IL-4, IL-13, CCL18) and CCL11, but higher baseline levels of CCL26, CD25/IL-2R alpha, IL-12p70, CCL17/TARC, TSLP and IL-5 (Fig 1). Furthermore, these patients demonstrated greater reductions in Th2 associated biomarkers (△IL-4, △IL-5, △IL-13, △TSLP, △CCL17/TARC, △CCL18/PARC) following treatment, aligning with better clinical outcomes (Supplementary Fig 4, available via Mendeley at https://data.mendeley.com/datasets/g6hvm2znjn/1).

**Fig 1:**
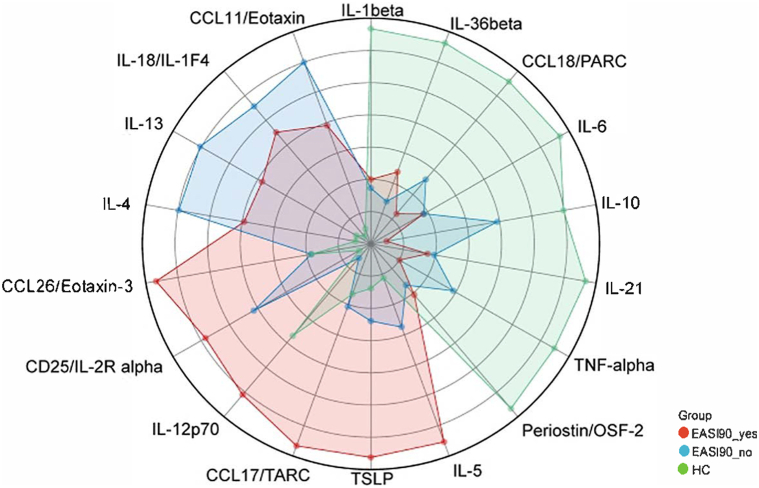
Radar chart illustrating the comparative expression profiles of baseline serum biomarkers in patients with atopic dermatitis (AD) and healthy controls (HC) subjects.

We further analyzed differentially expressed genes between patients with AD and HCs (fold change ≥4, FDR<0.05), and compared their expression changes post-treatment between EASI 90_yes and EASI 90_no patients. Genes that normalized toward HC levels only in EASI 90_yes patients were selected. Spearman correlation and ROC analyses identified 3 transcriptomic predictors: *WNT8B* (AUC = 0.764), positively correlated with clinical improvement in EASI%, SCORAD%, Sleep score, and NRS; while *KRT2* (AUC = 0.723) and *TTLL13P* (AUC = 0.756) showed negative correlations. Higher baseline *WNT8B* and lower *KRT2* and *TTLL13P* expression were associated with EASI-90 achievement (Supplementary Fig 5, *A*-*E*, available via Mendeley at https://data.mendeley.com/datasets/g6hvm2znjn/1).

These findings were validated in an independent cohort (*n* = 35) using RT-qPCR (Supplementary Table II, available via Mendeley at https://data.mendeley.com/datasets/g6hvm2znjn/1), which confirmed that EASI 90_yes patients had higher baseline *WNT8B* expression and lower levels of *KRT2* and *TTLL13P* (Fig 2), with AUC values of 0.795, 0.747, and 0.690, respectively (Supplementary Fig 5, *F*, available via Mendeley at https://data.mendeley.com/datasets/g6hvm2znjn/1). We further performed Youden’s index analysis to determine the optimal cut-off values for these biomarkers that maximize the sum of sensitivity and specificity in predicting EASI-90 response (Supplementary Table III, available via Mendeley at https://data.mendeley.com/datasets/g6hvm2znjn/1). The resulting cut-offs were 1.12 for *WNT8B*, 0.93 for *KRT2*, and 0.82 for *TTLL13P*, with corresponding sensitivities of 0.87, 0.73, and 0.40, and specificities of 0.70, 0.80, and 0.95, respectively.

**Fig 2:**
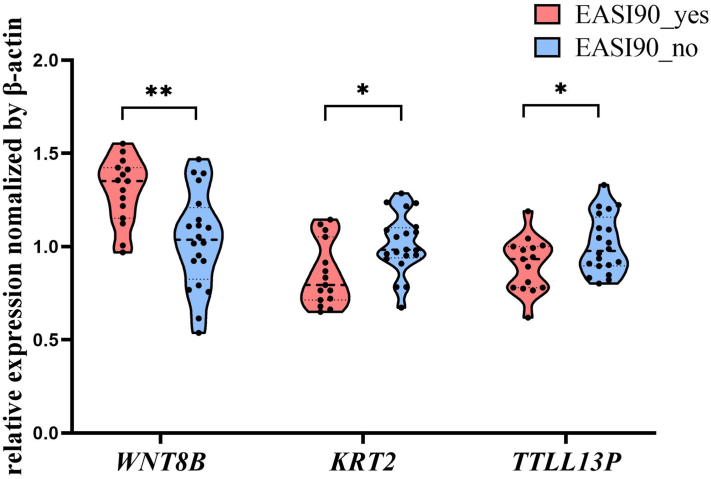
A comparison of the baseline expression levels of *WNT8B*, *KRT2*, and *TTLL13P* was validated between patients who achieved EASI-90 and those who did not.

In conclusion, our study identified transcriptional change (*WNT8B*, *KRT2*, and *TTLL13P*) as novel biomarkers predicting optimal response (EASI-90) of abrocitinib treatment in AD. However, achieving EASI-90 is a relatively high treatment target. Since patients often have individual needs and preferences, treatment goals should be established through shared decision-making between clinicians and patients.

## Conflicts of interest

Yao has received consultancy/speaker honoraria from Sanofi Regeneron, Pfizer, Abbvie, Novartis, LEO, and Pierre Fabre, and has participated as Principal Investigator in clinical trials sponsored by Sanofi Regeneron.
